# Thoracic foreign body management after penetrating chest trauma by chainsaw in the Amazon countryside: A case report

**DOI:** 10.1016/j.amsu.2021.103101

**Published:** 2021-11-23

**Authors:** João Lucas Miranda Fontelles, Messias Froes da Silva Júnior, Juan Eduardo Rios Rodriguez, Hafiza Gonçalves Alexandrino Regino, Estevan Criales Lopez, Júlia Fialho Cauduro, Héria Terezinha Rodrigues Brito Froes, Tatiana Minda Herculano Cattebeke, Arteiro Queiroz Menezes

**Affiliations:** a Department of General Surgery, Getúlio Vargas University Hospital (HUGV), Manaus, Brazil; b Department of Clinical Surgery, Medical School of Federal University of Amazonas (UFAM), Manaus, Brazil; c Medical School of Federal University of Amazonas (UFAM), Manaus, Brazil; d Medical School of FAMETRO University Center, Manaus, Brazil; e Oncology Control Center Foundation of Amazonas (FCECON-AM), Manaus, Brazil; f Department of Thoracic Surgery, Getúlio Vargas University Hospital (HUGV), Manaus, Brazil

**Keywords:** Chest trauma, Chainsaw, Foreign body, Case report

## Abstract

**Introduction:**

Accidents involving chainsaws are not uncommon in trauma care and may present as penetrating injuries with retention of a foreign object in the patient's chest. The current literature, however, does not present a consensus on the best way to approach these cases.

**Presentation of case:**

Male patient, 46-year-old man, born in Amazonas countryside, brought to the city of Manaus with a penetrating injury resulting from an accident with a chainsaw and retaining a 2cm sawtooth in his chest, six days after the event. After laboratory and imaging tests, as well as pre-operative preparation, an open thoracotomy was realized, the object was removed, and the patient was placed under a thoracostomy tube.

**Discussion:**

The diagnosis of chainsaw incidents is generally described in the literature as post-mortem, mainly due to the inappropriate use of the equipment. Surgical removal of a foreign body is indicated in most cases, except when it is peripheral or when there is some impossibility. Early surgical treatment benefits the patient, with lower mortality and morbidity.

**Conclusion:**

In view of the absence of consensus and guidelines to the approach of thoracic injury with foreign body retention, it is up to the surgeon to evaluate the best conduct in each case and according to the available resources.

## Highlights

• Chainsaw accidents are rare and have high mortality.
• The surgical removal of chest foreign bodies is indicated in most cases.
• The surgical management of these cases depends on the surgeon's experience.

## 1 Introduction

Accidents involving chainsaws, mainly chainsaws and circular saws, are generally described in the forensic literature and are associated with suicides or attempts, being considered rare exceptions [1]. The occurrence of these events is mainly attributed to the use of poor-quality

equipment or inappropriate use, according to the U.S. Consumer Product Safety Commission, 64,100 injuries associated with chainsaws were recorded in 2001 [2].

The presence of foreign bodies in the chest usually results from trauma with a firearm but can include several objects such as broken glass and metal splinters. Of the thoracic trauma with pulmonary involvement, about 30% have multiple injuries while 70% have injuries of varying length. The conduct in these cases, although still much debated, should always assess the risks of removing the object with the complications of leaving it on the patient [3]. In this report, we describe the case of a 46-year-old man who suffered a penetrating wound by a chainsaw tooth on his chest, similar to a gunshot wound, a very peculiar case. This case follows 2020 SCARE guidelines for reporting of cases in surgery [4].

### 1.1 Presentation of case

**Fig. 1:**
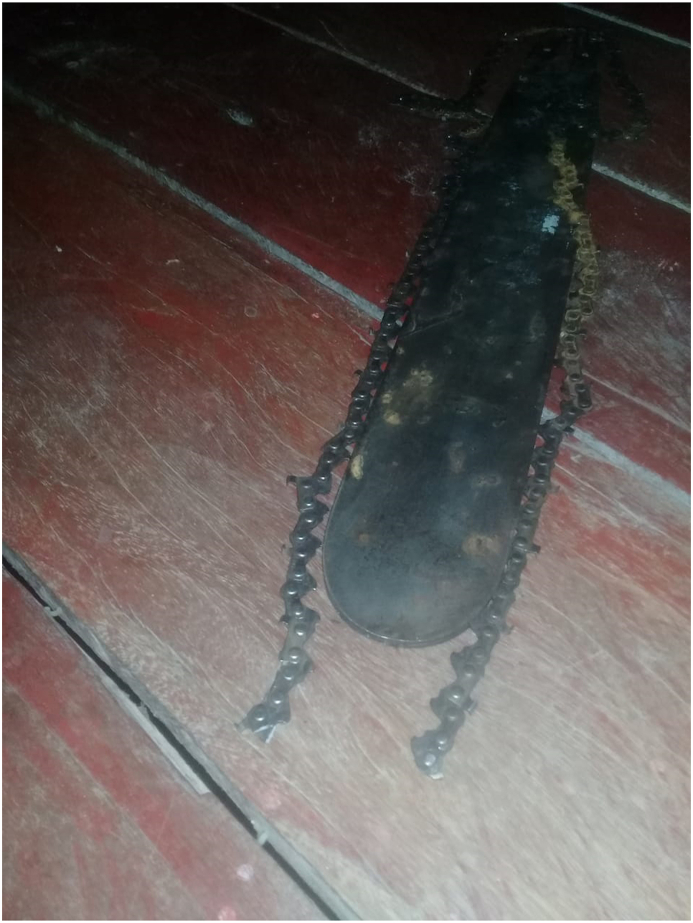
The broken chainsaw chain after the incident.

A 46-year-old male patient without a past medical history was transferred to our hospital due to a 6-day history of lung injury by a chainsaw tooth (Fig. 1). The patient reports that his son was cutting logs with a chainsaw approximately 20 m away, when the chain broke, and the projectile hit him in the left hemithorax. He was admitted at a local hospital on the same day, presenting dyspnea, left chest pain and a major bleeding through the entrance of the projectile. According to local reports brought with the patient, a chest X-ray showed no evidence of pneumo- or hemothorax. The chest was not drained. Digital exploration and synthesis of the skin was carried out. Due to the lack of computed tomography and thoracic surgeon at the local hospital, it was decided to transfer the patient to our hospital, a reference in thoracic surgery in the region. In addition, the difficulty of access and transportation of patients in the Amazon region, he was transferred to our hospital only on the sixth day after the injury.

**Fig. 2:**
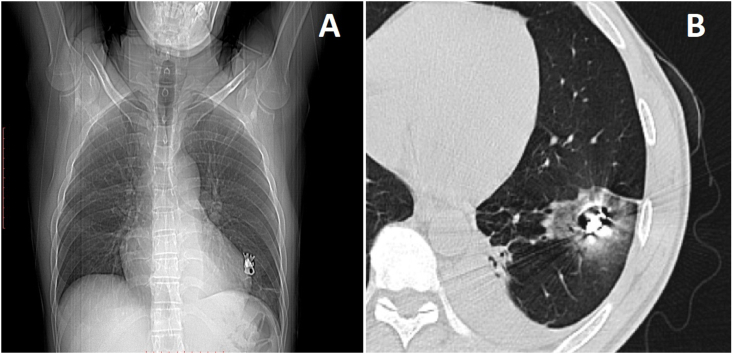
(A) Chest radiograph showing a 2-cm-long chainsaw tooth in the left hemithorax. (B) Computed tomography image showing a left-sided pleural effusion and a pulmonary injury of the left lower lobe.

At the admission to our hospital, the patient was hemodynamically stable, with no complaints. Physical examination revealed a bruise on his 7th left intercostal space, at the middle axillary line, healed, without exit wound or signs of infection. Clinically, the patient did not show signs of anemia, which was confirmed by laboratory examination - his hemoglobin concentration was 12.5 g/dL. His leukocyte count and serum C-reactive protein level were 6210/μL and 4.7 70 mg/dL, respectively, therefore indicating no residual inflammatory response. A chest radiograph showed a 2-cm-long chainsaw tooth in the left hemithorax and the computed tomography (CT) image showed a left-sided pleural effusion and a pulmonary injury of the left lower lobe due to a foreign body (Fig. 2). There was no evidence of hemo or pneumothorax, which was expected in a clinically stable patient with 6 days of evolution.

**Fig. 3:**
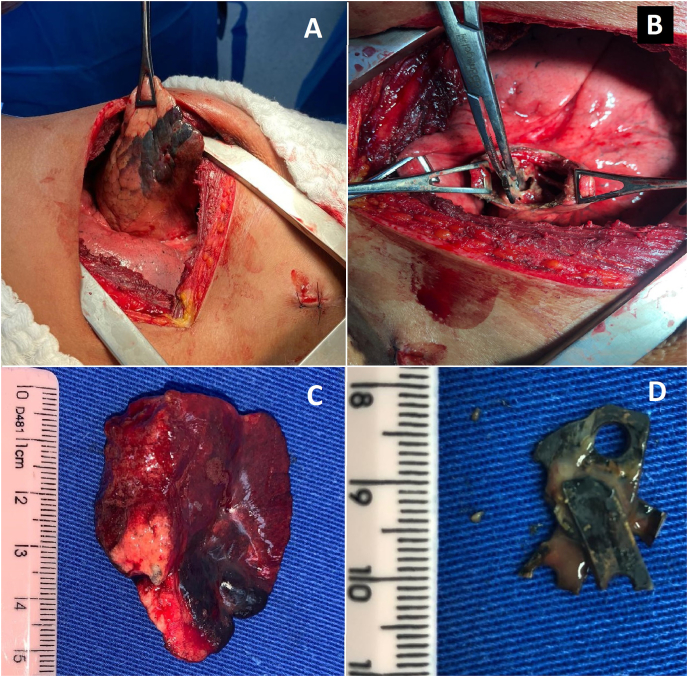
Surgical findings of the pulmonary injury: (A) Penetrating wound in the left lower lobe with signs of necrosis at the edges; (B) the foreign body was removed by pneumotomy; (C) the lacerated lung parenchyma was treated by partial pulmonary resection (D); the foreign body was a 2-cm-long chainsaw tooth.

The diagnosis of lung injury due to foreign body and the need for surgery were discussed with the patient. His written informed consent was obtained. Exploratory thoracotomy was performed one day after the admission to our hospital. Surgical findings showed low quantity of residual hemothorax, with no evidence of active bleeding, and the penetrating wound in the left lower lobe with signs of necrosis at the edges (Fig. 3). The foreign body was evidenced by palpation of the left lower lobe and then removed by pneumotomy, followed by pneumorrhaphy with 4–0 polyglactin 910 suture. The lacerated lung parenchyma was treated by non-anatomic segmentectomy and pneumorrhaphy with the same suture. After reviewing the chest cavity, without the presence of active bleeding or air leak, a chest tube nº 36 was placed. The patient had a good postoperative evolution. The chest tube was removed on fifth postoperative day and the patient was discharged on eighth postoperative day, satisfied and without further complications. The macroscopic findings showed that the foreign body was a 2-cm-long chainsaw tooth and the whole lacerated lung parenchyma was resected (Fig. 3C and D).

## 2 Discussion

The diagnosis of chainsaw incidents is generally described in the literature as post-mortem, mainly due to the inappropriate use of the equipment, poor product quality and suicide attempts [1,5]. Although still poorly studied, this type of accident is responsible for approximately 23,179 emergency visits per year in the United States [5]; in Brazil, this equipment is used mainly in the cutting of trees for logging where it accounts for a large part of occupational accidents, mainly in: legs (29%), feet (14%), trunk (12%), head (11%) and arms (9%) [6]. The reported case, although uncommon, is predicted by the Jorge Duprat Figueiredo Foundation, for Occupational Safety and Medicine (FUNDACENTRO) as a risk inherent to the use of a chainsaw, classified as a particle injury during the cutting of trees when using the chainsaw, being addressed as a penetrating chest trauma [7].

Thoracic trauma can be classified as closed or penetrating, depending on whether the pleural cavity is opened and often requires a quick diagnosis and immediate intervention, prioritizing the maintenance of ventilatory function and prevention of hypoxia. This approach is initiated by stabilizing the patient, following the algorithms described by the ATLS (Advanced Trauma Life Support) of the ABCDE of the trauma [8]. If stabilized, patients should undergo imaging tests before any intervention, with five indications for immediate intervention being described in the literature: cardiac tamponade; traumatic thoracotomy; tracheal, bronchial or esophageal injury; massive hemothorax and lesion of large vessels [9,10]. In the reported patient, no exams or surgical intervention were initially performed due to the deficiency of infrastructure, a common situation in the state of Amazonas countryside, which prolonged his diagnosis and treatment.

The hemothorax, one of the main complications of thoracic trauma, is suspected of every patient with blunt or penetrating trauma and should therefore be addressed with chest drainage. The 109 current literature indicates a surgical approach through the initial drainage of 1500ml or 200ml/hour in the first 4 hours [11]. However, the delay in approaching our patient with 6 days of evolution prevented the immediate resolution of the condition, configuring the residual hemothorax observed. Recent literature suggests that the presence of residual or retained hemothorax in the pleural cavity is a risk factor for other complications, including empyema and fibrothorax, and should be drained for two to three days, preferably by videothoracoscopy [12]. Due to impossibility in accessing video techniques in our hospital, we opted for traditional thoracotomy. The presence of a foreign body retained after chest trauma always requires assessment of the benefits of its removal weighted against the complications of leaving it in situ.

Some aspects to be evaluated for the conduct include the size of the object (usually greater than 1.5 cm) and the shape (usually irregular), a series of reports from the American Association of Trauma Surgery in the 1960s initially recommended the surgical approach for removal of the foreign body, but a few years later began to recommend only in cases refractory to conservative treatment [3,13]. Two studies concluded that occasionally, and in the absence of complications, surgical removal of the object is indicated if it is non-metallic or of large size and with sharp contours, or keep the body strange when it is peripheral or when there is anatomical impossibility to perform a removal [3,14]. Considering that the description of the sawtooth observed in our patient is compatible with the indication, we chose to remove it.

## 3 Conclusion

Considering the poor epidemiological data in the international literature and the lack of national data, it is difficult to establish national protocols or consensus for the treatment of these injuries. For the treatment of this case, we opted for the surgical approach, considering the fact that all foreign bodies in the chest must be surgically removed, except for those with a blunt or peripherally located shape. When comparing the saw tooth to a projectile capsule and assessing its ability to carry organic and inorganic debris, we will have the case, reported above, framed among the predictors for removal of foreign bodies proposed. To ensure the best approach for the patient presented, the conduct chosen by the team was to submit the patient to an exploratory thoracotomy to remove the foreign body, being submitted to an unregulated segmentectomy to remove devitalized lung tissue. It is up to the surgeon to decide, in agreement with the patient, the best treatment according to the case presented.

## Informed consent

Written informed consent was obtained from the patient for publication of this case report and accompanying images. A copy of the written consent is available for review by the Editor-in-Chief of this journal on request.

## Conflicts of interest

This report does not present conflicts of interest by the authors.

## Research registration

N/A.

## Guarantor

João Lucas Miranda Fontelles.

## Provenance and peer review

Not commissioned, externally peer reviewed.

## Please state any conflicts of interest

We do not have any conflicts of interests.

## Please state any sources of funding for your research

We do not have any funding source, this manuscript is just a case report, not a research.

## Ethical approval

As the manuscript is not a research study, we only have the patient consent for writing and others forms of publication. Also, the ethical approval for this case reports has been exempted by our institution.

## Fundings

No fundings available.

## Author contribution

João Fontelles, Juan Rodriguez, Hafiza Regino made contributions to conception and design. Estevan Lopez, Julia Cauduro, Héria Fróes and João Fontelles collected the patient details and wrote the paper. Tatiana Cattebeke, Arteiro Menezes, Messias da Silva Júnior made contributions to patient management. João Fontelles and Messias da Silva Júnior critically revised the article. All authors read and approved the final manuscript.

## Consent

Written informed consent was obtained from the patient for publication of this case report and accompanying images. A copy of the written consent is available for review by the Editor-in-Chief of this journal on request.

## Author contribution

João Fontelles, Juan Rodriguez, Hafiza Regino made contributions to conception and design. Estevan Lopez, Julia Cauduro, Héria Fróes and João Fontelles collected the patient details and wrote the paper. Tatiana Cattebeke, Arteiro Menezes, Messias da Silva Júnior made contributions to patient management. João Fontelles and Messias da Silva Júnior critically revised the article. All authors read and approved the final manuscript.

## Registration of research studies

The manuscript is a case report, not considered a formal research involving participants.

## Guarantor

João Lucas Miranda Fontelles.
